# Impact of pulsed electric field treatments on the growth parameters of wheat seeds and nutritional properties of their wheat plantlets juice

**DOI:** 10.1002/fsn3.1540

**Published:** 2020-04-05

**Authors:** Zahoor Ahmed, Muhammad Faisal Manzoor, Nazir Ahmad, Xin‐An Zeng, Zia ud Din, Ume Roobab, Abdul Qayum, Rabia Siddique, Azhari Siddeeg, Abdul Rahaman

**Affiliations:** ^1^ School of Food Science and Engineering South China University of Technology Guangzhou China; ^2^ Overseas Expertise Introduction Center for Discipline Innovation of Food Nutrition and Human Health (111 Center) Guangzhou China; ^3^ Institute of Home and Food Sciences Faculty of Life Sciences Government College University Faisalabad Faisalabad Pakistan; ^4^ Department of Human Nutrition The University of Agriculture, Peshawar Peshawar Pakistan; ^5^ Key Laboratory of Dairy Science Northeast Agriculture University Ministry of Education Harbin China; ^6^ Department of Chemistry Government College University Faisalabad Faisalabad Pakistan; ^7^ Department of Food Engineering and Technology Faculty of Engineering and Technology University Gezira Wad Medani Sudan

**Keywords:** bioactive compounds, growth parameters, nutritional values, pulsed electric field, wheat seeds

## Abstract

This study was designed to explore the impacts of the pulsed electric field (PEF; 2 to 6 kV/cm; a number of pulses 25 and 50) on wheat (*Tritium aestivum* L.) seeds before imbibition to improve the germination, growth, and their nutritional profile in juice form. It was observed that the PEF treatment at 6 kV/cm at 50 pulses increased water uptake, germination of seeds, and growth parameters of seedlings. A significant increase in total phenolic contents, DPPH, chlorophylls, carotenoids, soluble proteins, minerals, and amino acids in PEF‐treated seeds plantlets juice as compared to the untreated seeds plantlets juice was observed. The results indicate that the PEF may effectively stimulate the growth of the wheat kernels and positively affect their metabolism, optimize the nutrients, and enhance the strength of the wheat kernels plantlets.

## Graphical abstract

This study was designed to explore the impacts of the pulsed electric field (PEF; 2 to 6 kV/cm; a number of pulses 25 and 50) on wheat (*Tritium aestivum* L.) seeds before imbibition to improve the germination and growth and their nutritional profile in juice form. It was observed that the PEF treatment at 6 kV/cm at 50 pulses increased water uptake, germination of seeds, and growth parameters of seedlings. A significant increase in total phenolic contents, DPPH, chlorophylls, carotenoids, soluble proteins, minerals, and amino acids in PEF‐treated seeds plantlets juice as compared to the untreated seeds plantlets juice was observed. The results indicate that the PEF may effectively stimulate the growth of the wheat kernels and positively affect their metabolism, optimize the nutrients, and enhance the strength of the wheat kernels plantlets.

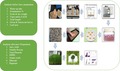

## 1 INTRODUCTION

Wheat kernel (*Tritium aestivum* L.) is a staple food grown worldwide. The human body needs carbohydrates, protein, vitamins, minerals, fibers, and antioxidants such as tocopherols, carotenoids, phenolic acids, phytic acid, flavonoids, tocotrienols, and phytosterols, which are commonly present in wheat kernels (Abdel‐Aal & Rabalski, 2008). Wheat plantlets aged 8–15 days, also known as a good source of bioactive compounds (ferulic, benzo(a)pyrene, caffeic, p‐coumaric acid, gallic, and syringic), minerals (iron, magnesium, and calcium), and vitamins (A, B, C, and E; Akbas et al., 2017). Wheat plantlets are nutrient‐dense widely consumed as raw juice immediately after production, as well as tablets and capsules at the commercial level. Wheat plantlets have various health beneficial properties such as antioxidant, neuroprotective, antidiabetic, and anticancerous (Mujoriya & Bodla, 2011).

Due to public awareness, the demands for functional foods and especially nutrient‐dense foods have been increased. Quality optimization techniques are necessary for the quality enhancement of functional foods (Ahmed et al., 2019; Manzoor, Ahmad, Ahmed, et al., 2019). Nowadays, the plantlets of different crops are considered an excellent source of bioactive compounds, which makes them the best functional food (Marton, Mandoki, Csapo‐Kiss, & Csapo, 2010). However; the market demand for plantlets is decreased due to the quality of plantlet metabolites. To overcome these problems, extrinsic compounds are used but they are evenly distributed to the kernels and could cause undesirable changes (Pérez‐Balibrea, Moreno, & García‐Viguera, 2011).

The pulsed electric field (PEF) is a nonthermal processing technology commercially used, due to power proficient, less energy consumption, and trifling processing technique (Rahaman et al., 2019; Siddeeg, Faisal Manzoor, Haseeb Ahmad, et al., 2019). This can alter the structural features of the agriculture products, through the assistance of bioactive metabolites discharge and/or improve the shelf life of foods, without the use of chemicals (Manzoor, Ahmad, Aadil, et al., 2019; Siddeeg et al., 2019; Toepfl, Mathys, Heinz, & Knorr, 2006). PEF has been considered to be a sublethal promising technique that enhances cell membrane permeability, the content of food material to the high voltage pulses of short repetition can cause temporary or everlasting pore formation in the cell membrane (Angersbach, Heinz, & Knorr, 2000; Siddeeg et al., 2019).

Seeds treatment by PEF has been revealed the growth response in different plants such as lettuce, barley, *Arabidopsis thaliana*, and many other plants. The inhibitory and stimulatory growth effects caused by PEF are based on the physiological state of seeds, electric field (EF) strength and intensity. Since the methods of seed germination and imbibition include a series of significant biochemical and physiological differences, the PEF technique could be applied to manipulate these processes (Leong, Burritt, & Oey, 2016). The plant cell has responses against stress condition, the common one is reactive oxygen species (ROS). The permeabilization of the cell membrane due to PEF usage is evidence of the production of ROS, the ROS lead to oxidative stress, which leads to an elevated level of antioxidants and many other metabolic components (Sabri, Pelissier, & Teissié, 1996). The aim of this study was to assess the impact of PEF on the seed. We monitored the plantlet's germination, growth, vigor index, water uptake, juice yield, soluble proteins, chlorophyll, amino acids, minerals, and bioprotective properties.

## 2 MATERIALS AND METHODS

### 2.1 Seeds selection

The nongenetically modified (NON‐GMO) wheat (*Tritium aestivum *L.) kernels were purchased from the Cereal Crop Research Institute (CCRI) Nowshehra, Pakistan from a single batch for the regular size, shape, and color. Debris and damaged seeds were removed from the experimental sample.

### 2.2 Pulsed electric field (PEF) treatment

**FIGURE 1:**
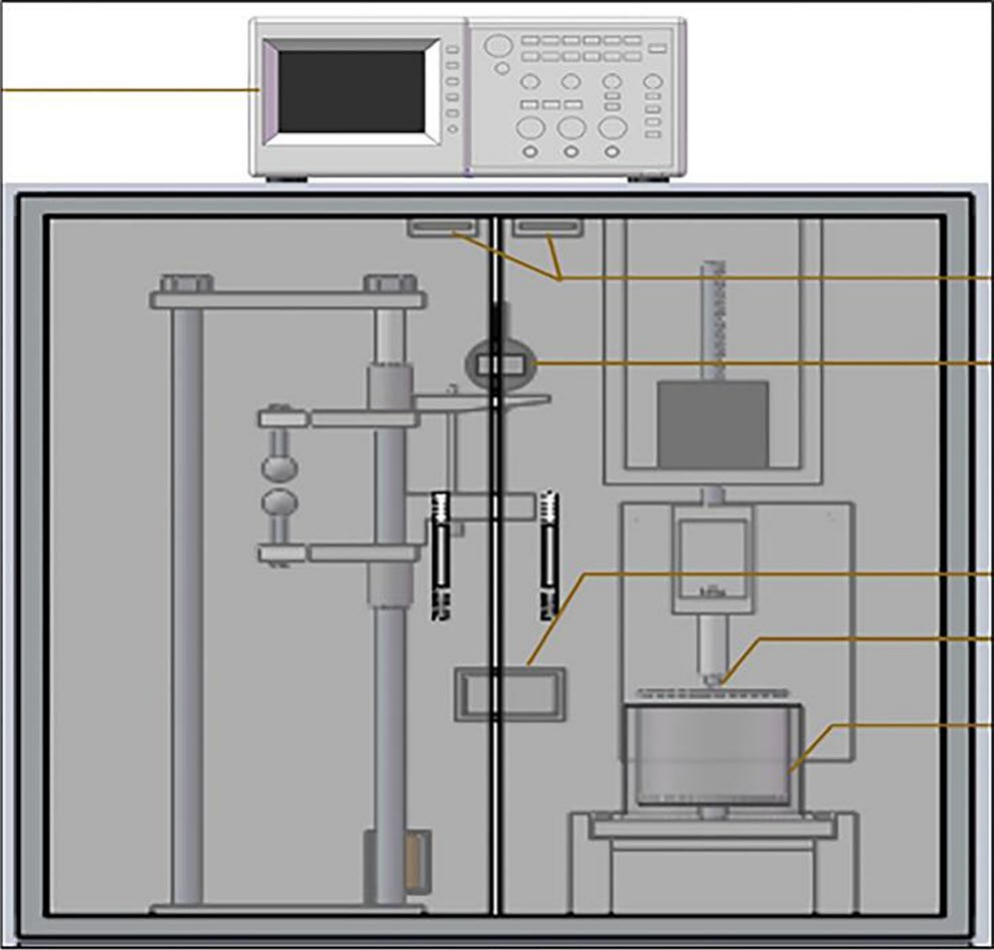
The Schematic diagram of PEF extraction s system

Wheat kernels were not immersed in water before treatment, they were divided into groups according to the PEF‐treated and untreated kernels. For the treatment, a laboratory‐scale PEF (EX‐1900, SCUT, PEF‐Team) was used (Figure 1). The treatment chamber has 10 cm of length and 11 cm of width with two corresponding electrodes. The chamber was filled with 40 g wheat kernels and 160 g distilled water, submerged the kernels and facilitated the equal EF strength in the course of PEF treatment. In this experiment, we used different EF strength of 2, 4, and 6 kV/cm for (25 and 50 pulses) with a pulse width of 100 μs at a stable frequency of 1 Hz. The temperature was maintained for all the treatments (<30°C).

For the calculation of voltage and specific energy used, following formulae 1–2 were used and values are presented in Table 1.

(1) $$E=10U_{m}/d$$

where *d* is the resistance between lower and upper electrodes of the sample solution container in cm; *U* is oscilloscope (kV) to estimate the voltage (10 load resistances of the electrodes at both ends of the sample solution.

(2) $$W_{\text{specific}}=W_{\text{pulse}}\times n/m\;\left(\text{kJ/kg}\right)$$

where *n* is the number of pulses, *W* is a pulse as per pulse energy, and *m* is the mass of the sample.

**TABLE 1:** The energy input (kJ/kg) of the pulsed electric field in the different treatment conditions

Electric fields strength (kV/cm)	Number of pulses (*n*)	Specific energy (kJ/kg)
2	25	1.5
	50	2.5
4	25	2.5
	50	5.0
6	25	3.7
	50	7.5

### 2.3 Water uptake index

Wheat kernels water uptake was estimated by weighing seeds before treatment. The seeds (3.20 g) were measured by almost 100 seeds/treatment. PEF treatment with different intensities (2, 4 and 6 kV/cm) and different numbers of pulses (25 and 50) was applied. The water uptake determined gravimetrically at the scheduled time, the surface water removed with tissue paper. The electric balance (error of ±0.1 g) weighed the kernels and then returned to the dish and reweighed at the scheduled time. Kernels imbibed for 3, 6, 20, and 24 hr at 20 ± 2°C in the dark condition. The increase in the weight of the kernel stated the amount of water uptake.

### 2.4 Cultivation for germination rate, leaf area, sencent, and green leaf

#### 2.4.1 Cultivation

The cultivation was performed in the petri dish at filter paper, taking 25 kernels/sample. The dish was filled with 3 ml of deionized water and left for 3 days. On the third day, 5 ml of deionized water was added and left for three days more, for 6 days cultivation in dark at 22°C.

#### 2.4.2 Germination rate

The germination rate was estimated as a ratio between a number of germinated kernels and the total number of kernels per dish. The kernels were determined as germinated when the length of a 1 mm radical sprout from the kernel appeared. For the calculation of the germination rate, following equation was used;

(3) GP(\% )=seedsgerminated/totalseeds×100

#### 2.4.3 Vigor index

The plantlet's length was measured by selecting 10 shoots randomly with mm scale. The germinated kernels shoots were measured freshly, and for drying purpose packed into aluminum foil separately at 60°C for three days with the known weight. The weight of one seedling fresh and dry plantlet was measured. The data were used vigor index calculation:

(4) $$\text{Vigor}\;\text{index}\;A=\text{Germination}\;\left[\%\right]\;\times \;\text{Fresh weight}\;\left[\text{mg}\right]/100$$

(5) $$\text{Vigor}\;\text{index}\;B=\text{Germination}\;\left[\%\right]\;\times \;\text{Dry}\;\text{weight}\;\left[\text{mg}\right]/100$$

#### 2.4.4 Leaf area, sencent, and green leaf

For the determination of sencent, and green, selected 14 leaves and divided as green (healthy) or senescent (physiological aging) as described by Chanda and Singh (2002). The leaf area measurement was done with the help of a simple following formula:

(6) $$Y=0.75\;LW\;\left({\text{cm}}^{2}\right)$$

where *Y*, *L*, and *W* are leaf area, length (cm), and width (cm), respectively.

### 2.5 Germination for Juice preparation

Wheat organic seeds of variety (Inqilab‐91) were procured from CCRI Nowshehra, Pakistan. The reason for choosing this variety was highly nutritive value among the other 16 varieties available in Khyber Pakhtunkhwa, Pakistan. The wheatgrass was grown inside the laboratory where sufficient light and airflow were present. Warm water was used to wash the wheat seeds for the removal of microbial load and undesirable adhered material. Wheat seeds were then soaked for 24 hr in distilled water, then drained. After that, seeds were wrapped in a muslin cloth and left for sprouting at least for 12 hr. Grains were spread on soil trays, and the water was sprinkled every day. Wheat plantlets were cut with scissors after 14 days, 2 cm above the soil. Wheat plantlets juice was extracted using specifically designed manual juice extractor suitable being fibrous in nature. It pressed the plantlets employing centrifugal force; manual plantlets juicers also prevent oxidation and heat. Then, wheat plantlets juice was extracted by the masticating process after washed and air‐dried.

### 2.6 Soluble protein content

The plantlet juice was accurately measured and mixed according to the ratio of weight (g): volume (ml) = 1:9. The solution was centrifuged for 10 min at 10000 *g*, then the supernatant was removed. Take 1 ml of protein (Coomassie bright blue method No. A045‐2) reagent, 0.5 ml of the standard mixed well and left for 10 min then measured the absorbance at 595 nm as a standard value, the same procedure was used for absorbance measurement of samples.

### 2.7 Pigments content estimation

The plantlets were used to estimate the pigments. The sample of average 0.5 g weight was homogenized with sand, MgCO_3_, and 80% acetone. The solution was filtered and diluted to the absorbance range. The chlorophyll a, chlorophyll b, and carotenoids were measured by spectrophotometer at 664, 648, and 470 nm accordingly (Lichtenthaler, 1987)**.**

### 2.8 Determination of total phenolic

The total phenolic contents of wheat plantlet juice were determined by a method proposed by Manzoor, Zeng, et al. (2019) with little modification. Briefly, 1 ml of Folin–Ciocalteu reagent was added to a 250 µl of wheat plantlet juice. After the addition of 2 ml sodium carbonate (20%), the mixture was mixed well and then left for 12 min at ambient temperature in a dark. Then, spectrophotometric (UV‐1800, SHIMADZU) absorbance was measured at 760 nm. A suitable calibration curve was prepared before analyzing the sample using a standard solution (1 mg/ml) of gallic acid, and the results were expressed as µg of gallic acid equivalents (GAE) per ml of sample.

### 2.9 2,2‐diphenyl‐1‐picrylhydrazyl (DPPH) activity

The DPPH activity of the wheat plantlets juice was measured according to the method of Manzoor, Zeng, et al. (2019). Take 250 µl of each sample and then mixed with 50 µl of 1 mM DPPH solution prepared in methanol. After that incubated for 30 min in dark at room temperature. Spectrophotometric absorbance was measured at 517 nm against the blank. DPPH (%) was calculated by the following equation;

(7) $$\text{Scavenging}\;\text{effect}\;\%=\left[{\text{Abs}}_{\text{control}}-{\text{Abs}}_{\text{sample}}\right)]/{\text{Abs}}_{\text{control}}\times 100$$

The DPPH percentage values were plotted against the Trolox concentration and the antioxidant capacity measured from the linear equation.

### 2.10 Free amino acids

The free amino acids of treated seeds plantlets were distinguished by an A300 amino acid analyzer (membraPure GmbH). The software iPeak and iControl (membraPure) were used. In brief, 4 ml of each sample supplemented with 3, 5‐dinitrosalicylic acid (1 ml) was stored for 1 hr at 4°C to invert the protein and centrifuged twice at 10000 *g* for 15 min. Diluents comprising acetic acid, formic acid, trifluoroacetic acid lithium acetate, and ethanol to the final amino nitrogen of 0.008%–0.01% were used to dilute the supernatant. Liquid chromatography with a column (TS263, membraPure) was used to separate the amino acids and detected by ninhydrin reaction, and then the absorbance was recorded at 570 and 440 nm for proteins. Each amino acid concentration was measured using external standards (Sigma–Aldrich). All values were expressed in mg/100 ml.

### 2.11 Mineral elements

Mineral contents of wheat plantlets juice were estimated using atomic absorption spectroscopy (Hitachi Z, 2000, Polarized, Zeman) by the method of Aadil et al. (2015). One milliliter of wheat plantlets juice sample was poured into a Teflon vessel. After that digested on a microwave work station with 1 ml of 30% H_2_O_2_ and 7 ml of 65% HNO_3_ and acid digested sample was diluted. A hollow‐cathode lamp was used as the radiation source. For each mineral compound, the standards were prepared within the rage of mineral elements concentration contained in the sample. The results were described in mg/L of samples.

### 2.12 Statistical analysis

All experiments in the present study were carried out in triplicate. The experimental data were statistically analyzed using SPSS software version 24 (IBM SPSS Statistics). In this research, *p*‐value < .05 was considered statistically significant.

## 3 RESULTS AND DISCUSSION

### 3.1 Water uptake

**FIGURE 2:**
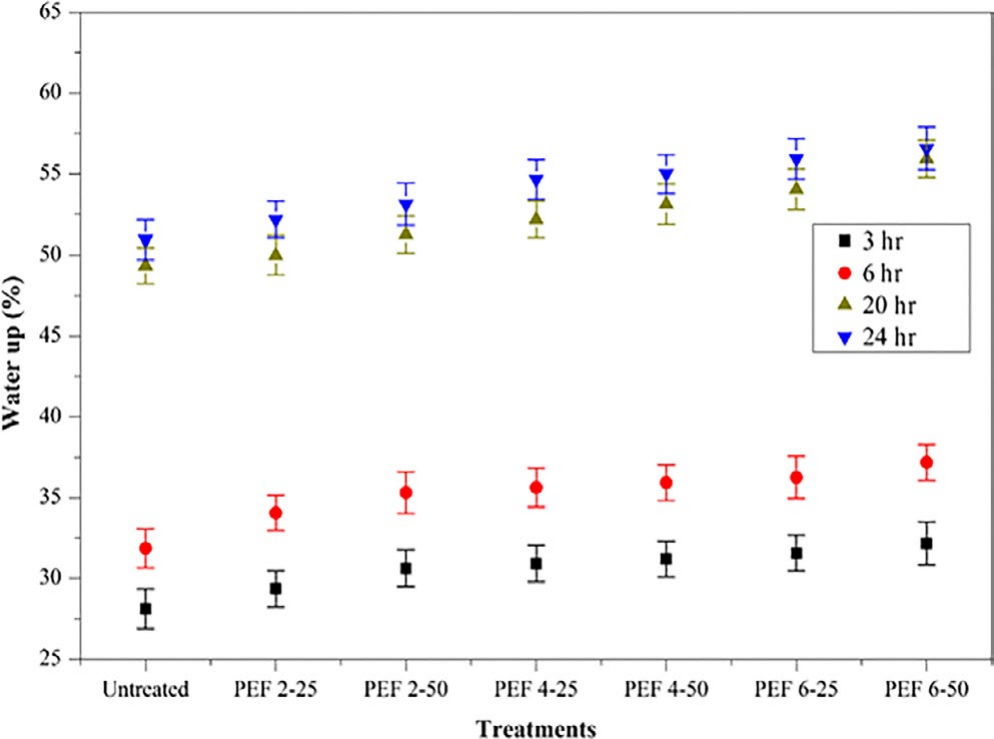
Impact of PEF treatment (2, 4, and 6 kV/cm, with 25 and 50 pulses) on the water uptake capacity of wheat seeds during the 3, 6, 20, and 24 hr. Values are shown as mean ± *SD* (*p *< .05). PEF 2‐25, treated at 2 kV/cm with 25 pulses; PEF 2‐50, treated at 2 kV/cm with 50 pulses; PEF 4‐25, treated at 4 kV/cm with 25 pulses; PEF 4‐50, treated at 4 kV/cm with 50 pulses; PEF 6‐25, treated at 6 kV/cm with 25 pulses; PEF 6‐50, treated at 6 kV/cm with 50 pulses

The results of water uptake after the treatment of seeds at different EF intensities (2, 4, and 6 kV/cm) and the number of pulses (25 and 50) are shown in Figure 2. The rapid water uptake leads to stimulate faster germination and strengthens growth. The maximum impact of PEF treatment was inspected in seeds treated at 6 kV/cm with 50 pulses; the water uptake was 56.56% at 24 hr. The water uptake in untreated seeds was 50.93% at 24 hr. It was observed that by increasing the EF intensities and the number of pulses, the water uptake capacity was increased. The highest water uptake was observed during the first 3 hr after the PEF treatments (Figure 2). The accretion in the water uptake capacity of PEF‐treated seeds at 6 kV/cm with 50 pulses guides to enhance the ability to absorb nutrients that assist plantlet growth and strength (Bhardwaj, Anand, & Nagarajan, 2012). For germination, the water uptake is the initial step that activates the enzymes which affect the catabolism of seed reserves. Rapid hydration of seeds leads to enhance amylase and protease activity during seed germination. These enzyme's activity was reported in cucumber seeds with magnetopriming (Bhardwaj et al., 2012).

### 3.2 Germination and juice yield

**FIGURE 3:**
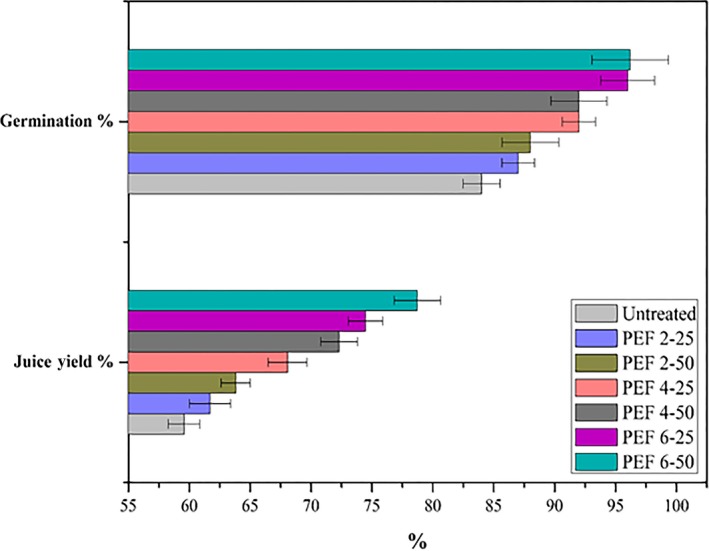
Impact of PEF treatment (2, 4, and 6 kV/cm, with 25 and 50 pulses) on the germination (%) and juice yield (%) of wheat seed plantlets. Values are shown as mean ± *SD* (*p* < .05). PEF 2‐25, treated at 2 kV/cm with 25 pulses; PEF 2‐50, treated at 2 kV/cm with 50 pulses; PEF 4‐25, treated at 4 kV/cm with 25 pulses; PEF 4‐50, treated at 4 kV/cm with 50 pulses; PEF 6‐25, treated at 6 kV/cm with 25 pulses; PEF 6‐50, treated at 6 kV/cm with 50 pulses

The germination percentage of seeds after different PEF treatments is presented in Figure 3 after 24 hr. Our results showed that the PEF treatment at 6 kV/cm increased the rate of germination as compared to the other treatments and untreated seeds. The seed's germination inclination reveals the relativity with the water uptake data that more hydration led to faster germination. The stimulating effect of PEF 6 kV/cm on germination was probably due to hydration and the bran stress associated with EF strength. The positive outcome of PEF on seeds germination was already described for various plant species (Costanzo, 2008). PEF excites metabolic activities and respiration through the generation of O_2_ during the catalase scavenging of H_2_O_2_. This can damage the seed coat and help the water diffusion or may oxidize the inhibitors (Barba‐Espin et al., 2010). The PEF inscribed some positive influences on the reactive species, like OH or O_2_, NO_3_, NO_2_, and NO which stimulate germination in a different type of plant species and activate dormant seeds (Finch‐Savage & Footitt, 2012). The impact of PEF on the germination rate depends upon the plant species and in vivo or in vitro experiments. Earlier, Lindsay et al. (2014) noticed nonsignificant variations in the germination of marigold, tomato, and radish, grown in soil pots after the same treatments. Zhang, Rousseau, and Dufour (2017) stated a 50% increase in the germination rate of lentil seeds. In these consequences, the PEF for different plant species could respond in quite different modes.

Juice yield was estimated on the bases of the same wheat plantlet's weight for all treatments (Figure 3). The obtained yield was varied with different PEF intensities; a significant increase was observed as untreated (59.57%) to the highest PEF intensity‐treated seeds (kV/cm) (78.72%). The PEF treatments increased the juice yield from 2.13% to 19.15%. This is possibly associated with more water uptake, fast germination associated large leaf area, resulting in strength of the plantlet, and finally increased the juice yield. Earlier, Eshtiaghi and Knorr (2000) stated that a 4.5% increment in juice yield treated with PEF.

### 3.3 Growth parameters

The important growth factors of wheat plantlets after 6 days of seed growth, such as the weight, length, and vigor indices A and B, are presented in Table 2. Our results indicate that the most significant effect of PEF treatment was observed at 6 kV/cm with 50 pulses and the length of plantlets increased from 3% to 9% for untreated to the highest intensity‐treated seeds. The fresh weight of plantlets increased from 8% to 42% for untreated to the highest intensity treatment. The PEF influence was considered as the change in the fresh and dry weight of the plantlets. The dry weight represents the biomass and indicates fewer errors as compared to fresh weight. The PEF induces the pore formation in seed coat and affects the metabolic process, it is an efficient technique for the transfer of external materials inside the cell, as well as for maintaining good simulative effects on plant intracellular organelles (Kandušer & Miklavčič, 2009). Therefore, the highest intensity of PEF treatment causes significant differences in the results.

**TABLE 2:** Growth parameters of PEF‐treated and untreated wheat seed plantlets

Parameters	Untreated	PEF 2‐25	PEF 2‐50	PEF 4‐25	PEF 4‐50	PEF 6‐25	PEF 6‐50
Fresh weight (mg/seedling)	80.2 ± 0.58^e^	88.5 ± 0.71^d^	92.1 ± 0.61^d^	101.3 ± 1.10^c^	105.7 ± 0.99^c^	114.4 ± 1.27^b^	122.6 ± 1.72^a^
Dry weight (mg/seedling)	18.60 ± 0.27^de^	20.46 ± 0.73^d^	21.40 ± 0.48^d^	23.48 ± 1.04^bc^	23.90 ± 0.47^bc^	25.51 ± 0.55^b^	27.37 ± 1.68^a^
Total length (cm/seedling)	9.00 ± 0.40^e^	9.3 ± 0.07^d^	9.5 ± 0.25^c^	9.7 ± 0.24^b^	9.7 ± 0.42^b^	9.8 ± 0.07^a^	9.9 ± 0.34^a^
Vigor index A	67.2 ± 3.23^g^	75.65 ± 0.99^f^	80.96 ± 1.22^e^	92.92 ± 134^d^	96.60 ± 3.23^c^	109.44 ± 1.22^b^	117.12 ± 2.70^a^
Vigor index B	15.62 ± 0.45^cd^	17.80 ± 0.48^c^	18.83 ± 0.23^c^	21.60 ± 1.26^b^	21.98 ± 1.15^b^	24.01 ± 0.53^a^	25.92 ± 1.96^a^
Soluble protein (mg/g)	8.94 ± 0.23^c^	8.98 ± 0.19^c^	9.46 ± 0.29^b^	9.51 ± 0.17^b^	9.62 ± 0.14^b^	9.74 ± 0.16^b^	10.02 ± 0.21^a^
TPC (Gallic acid/100 ml)	280.12 ± 1.23^e^	291.23 ± 2.32^d^	299.45 ± 3.12^c^	312.32 ± 1.57^b^	315.65 ± 2.76^b^	328.88 ± 2.14^a^	332.11 ± 1.67^a^
DPPH (Trolox µM)	1,314.4 ± 3.13^e^	1,339.7 ± 3.42^d^	1,345.8 ± 4.16^c^	1,362.2 ± 2.51^b^	1,369.5 ± 4.46^b^	1,382.9 ± 3.24^a^	1,390.3 ± 3.62^a^

Note

Outcomes with distinct letters in the same row (a–g) are significantly (*p* < .05) different from each other. PEF 2‐25, treated at 2 kV/cm with 25 pulses; PEF 2‐50, treated at 2 kV/cm with 50 pulses; PEF 4‐25, treated at 4 kV/cm with 25 pulses; PEF 4‐50, treated at 4 kV/cm with 50 pulses; PEF 6‐25, treated at 6 kV/cm with 25 pulses; PEF 6‐50, treated at 6 kV/cm with 50 pulses.

### 3.4 Growth parameters (leaf area, green, and senescent leaves)

**FIGURE 4:**
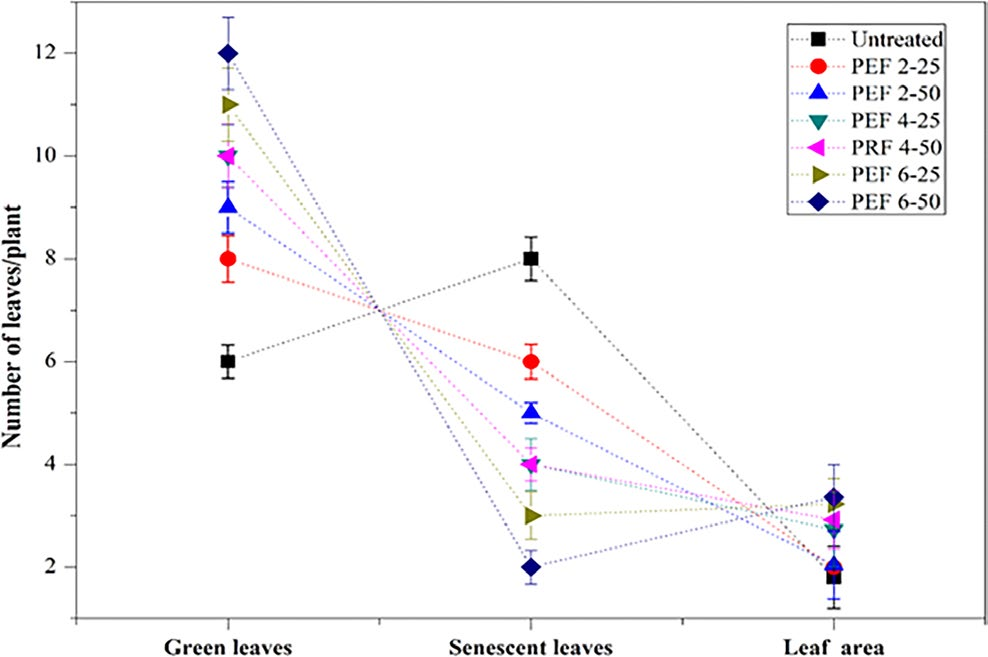
Area of the second fully developed leaf and number of green and senescent leaves of PEF‐treated (2, 4, and 6 kV/cm, with 25 and 50 pulses) and untreated wheat seeds plantlets. Values are shown as mean ± *SD*. Values are shown as mean ± *SD* (*p* < .05). PEF 2–25, treated at 2 kV/cm with 25 pulses; PEF 2‐50, treated at 2 kV/cm with 50 pulses; PEF 4‐25, treated at 4 kV/cm with 25 pulses; PEF 4‐50, treated at 4 kV/cm with 50 pulses; PEF 6‐25, treated at 6 kV/cm with 25 pulses; PEF 6‐50, treated at 6 kV/cm with 50 pulses

The results of leaf area, number of green and senescent leaves in untreated seed plantlets, and PEF‐treated plantlets are shown in Figure 4. The PEF‐treated seeds at 6 kV/cm with 50 pulses had significant leaf area (46.47%) as compared to untreated and other low‐intensity‐treated samples. In the case of greener and less senescent leaves, PEF treatment at 6 kV/cm with 50 pulses had a significant effect. The early aging of plantlets caused by the deficiency of many elements needed for rapid growth (Kučerová, Henselová, Slováková, & Hensel, 2019). The results show a significant impact on the growth; however, it can optimize the density of nutrients in the plantlets, and it can be further enriched with increment in the cultivation time for more than 15 days. The results indicated that PEF treatment enhances nutrient intake, such as nitrogen for the integration of biomolecules yield and active photosynthesis. Earlier Maniruzzaman, Sinclair, Cahill, Wang, and Dai (2017) described that the H_2_O_2_ and nitrate produced in water by electric discharge caused a beneficial impact on plant growth, increase biomass, and leaves length in wheat plantlets. The PEF experimental procedure can be declined the plant tissue, which gives more compounds beneficial for growth parameters (Ersus, Oztop, McCarthy, & Barrett, 2010).

### 3.5 Photosynthetic pigments

**FIGURE 5:**
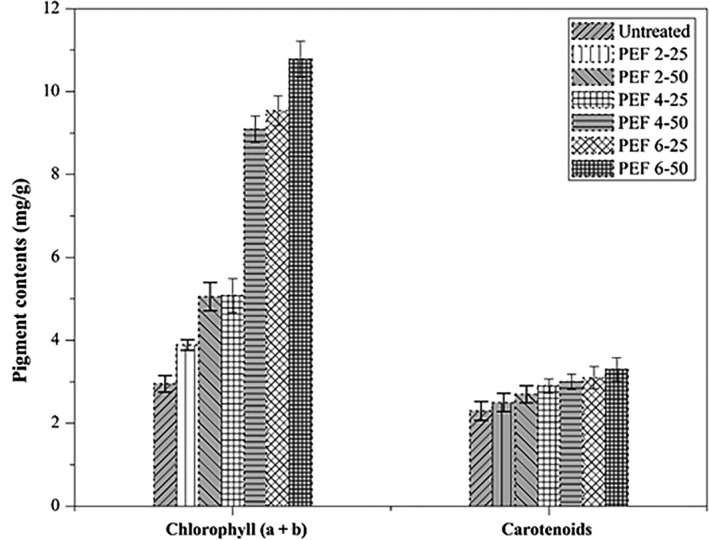
Chlorophylls (a + b) and carotenoids content in PEF‐treated (2, 4, and 6 kV/cm, with 25 and 50 pulses) and untreated wheat seed plantlets. Values are shown as mean ± *SD* (*p* < .05). PEF 2‐25, treated at 2 kV/cm with 25 pulses; PEF 2‐50, treated at 2 kV/cm with 50 pulses; PEF 4‐25, treated at 4 kV/cm with 25 pulses; PEF 4‐50, treated at 4 kV/cm with 50 pulses; PEF 6‐25, treated at 6 kV/cm with 25 pulses; PEF 6‐50, treated at 6 kV/cm with 50 pulses

The chlorophylls and carotenoids were increased with the PEF treatments; the stress may promote respiration or initiate metabolic reactions, which might lead to polyphenolic accumulation in plantlets. Many researchers reported that stress increases metabolites (Cantos, García‐Viguera, de Pascual‐Teresa, & Tomás‐Barberán, 2000). The contents of chlorophyll and carotenoids of PEF‐treated seed plantlets are displayed in Figure 5. Results indicate that the increase in PEF treatment led to increase chlorophyll and carotenoids content. In the case of total chlorophyll contents, a significant increment was observed in PEF4‐50, PEF6‐25, and PEF6‐50. The increased level of chlorophyll leads to an increased rate of photosynthesis and overall plant metabolism (Ramalho, Marques, Semedo, Matos, & Quartin, 2002). The increase in increment shows that oxidative stress increased in plant cells. On the other hand, carotenoids contents were gradually increased by increasing the PEF intensities and the highest increment increase (34%) was observed in PEF6‐50 treatment as compared to the untreated seed plantlets. After the PEF treatments, the softening of the plant tissue and protein denaturation could help to release carotenoids. The chloroplast accumulates protein complexes with carotenes incorporation which restricts the release of carotenoids (Sánchez, Baranda, & de Marañón, 2014).

### 3.6 Total phenolic component (TPC) and antioxidant activity (DPPH)

PEF‐treated seeds wheat plantlets were observed to be rich in total phenolic contents in 14‐day‐old wheat plantlets (Table 2). The phenolic compounds in plantlets grown after the PEF treatments were significantly (*p *> .05) increased. The PEF puts stress on seeds that affect the content of metabolites in organisms, such as phenolic compounds. Germination condition and PEF stress caused changes in the total content of phenolic compounds in embryos of wheat grain. The results of some previous and the present studies showed that the application of the PEF technique to the hydrated seeds can change the stress responses and growth of the resultant seedlings. PEF treatments of wheat seeds can create sustained and significant variations in seedling metabolism. Hence, the PEF treatment of seeds possibly has a scale of practical application in sprouts production with modified nutritional characteristics. We found that the contents of some metabolites were changed after PEF treatment, and these differences support the hypothesis that PEF penetrates into the caryopses and affects metabolic processes, because of the direct PEF treatment of the seed coat and influence on cells inside the seed. As we know the shoots of wheatgrass are good sources of phenolic and chlorophyll, all with strong antioxidant features, we observed whether the juice of wheat plantlets from PEF‐treated seeds, with improved antioxidant metabolism, had a better antioxidant ability than the juice of wheat plantlets from the untreated seeds. The increase in phenolic and antioxidant activity of wheat plantlets from PEF‐treated seeds might be due to the interaction between PEF and cells, which causes the cell wall fracture, DNA damage, change enzymatic activity, protein structure, and excite natural signal (e.g., opening the calcium channel and stimulation of growth components). Earlier, Zhang and Björn (2009) and Petit et al. (2009) reported these kinds of effects after the UV treatment to different plant seeds.

### 3.7 Soluble protein

Wheat is widely grown as a significant crop in the world; the one reason is its high amount of protein. The soluble protein plays a vital role in the growth of the plants and a substantial part of many plant enzymes that indicted the metabolism. The uncombined proteins in the cell and or organelle membrane represent the soluble protein (Sinha, 2004). The PEF‐treated wheat seeds plantlets and their protein concentrations are presented in Table 2. The significant increase (16.76%) in soluble protein was observed in PEF‐treated wheat seed plantlets with PEF6‐50 as compared to untreated wheat seed plantlets. Earlier, Toepfl, Heinz, and Knorr (2006) stated that the increase in soluble protein was due to the voltage stress or voltage‐sensitive channel, the channel activated at a deficient level of voltage, in comparison to a critical transmembrane potential which might open due to electrical damage, the easy availability of nutrients caused the increment. Protein synthesis was found to be stimulated in electroporated plant protoplasts of *Dactus carotta* L.,* Nicotiana tabacum *L., and *Beta vulgaris *L. after a single rectangular pulse of 400 μs at 1,700, 1,200, and 1,000 V/cm, respectively (Joersbo & Brunstedt, 1990).

### 3.8 Free amino acids profile

The contents of the free amino acid of untreated and PEF‐treated wheat seed plantlets are shown in Table 3. The results show that there was a significant increase in the total number of free amino acids of wheat seed plantlets with increasing the EF strength from 2, 4, and 6 kV/ cm and numbers of pulses (25 and 50), but individually most of the amino acids were reduced. The highest increase in total free amino acids (21.7%) was observed in PEF6‐50. The observed variation in the concentration of amino acids during a sprouting stage can be associated with the activation of 2nd cycle of metabolism needed for shoots and roots development as (a) storage protein hydrolysis improved the activity of protease (Khan, Verma, & Sharma, 2010), (b) protein mobilization from cotyledons to the newly developed roots and shoots for the growth of sprouts (Rodríguez, Frias, Vidal‐Valverde, & Hernández, 2008), (c) amino acids biosynthesis genes metabolism activation in the initial stage of sprouting resulted in the synthetic activity of these amino acids (Kanmegne, Mbouobda, Temfack, Koff, & Omokolo, 2010; Ruuska, Girke, Benning, & Ohlrogge, 2002). The broad variability in amino acid levels such as alanine, proline, arginine, and leucine in the wheat seed endosperm is fair to believe that this indicates active metabolism. It looks sensible to propose that these variations in the amino acid metabolism of the treated seeds may be a result of PEF. PEF caused permeabilization and the biological cell experiences distinct mutations when bared to high voltage electric pulses. Results of these electrical pulses on structural modifications and cell membrane break down depending on the pulse duration, EF strength and number of pulses. Earlier, the protein synthesis was observed to be stimulated in electroporated plant protoplasts after a single rectangular pulse of 400 μs at 1,700, 1,200, and 1,000 V/cm (Joersbo & Brunstedt, 1990). After electroporation, the cultivation time with the change in amino acid profile was seen to be dependent on the employed field strength. Present research work describes that PEF treatment affects the biological systems, which proposes new feasibilities to targeted changes in functional food properties. When employing PEF treatments, it may be used as an external stimulus for the initiation of stress reactions in plant systems supporting the creation of secondary metabolites as a plant response on strained conditions.

**TABLE 3:** Amino acids (mg/100 ml) profile of PEF‐treated and untreated wheat seed plantlets

Amino Acids	Untreated	PEF 2‐25	PEF 2‐50	PEF 4‐25	PEF 4‐50	PEF 6‐25	PEF 6‐50
Asp	17.86 ± 0.21d	30.76 ± 0.16a	23.71 ± 0.14c	26.32 ± 0.11b	32.66 ± 0.09a	25.57 ± 0.13b	24.95 ± 0.18b
Thr	BDL	BDL	BDL	BDL	BDL	BDL	BDL
Ser	BDL	BDL	BDL	BDL	BDL	147.58 ± 1.12b	160.77 ± 1.32a
Glu	BDL	BDL	BDL	BDL	BDL	1.124 ± 0.07a	BDL
Gly	14.94 ± 0.11b	15.42 ± 0.16a	14.41 ± 0.08b	14.75 ± 0.07b	15.07 ± 0.09a	7.18 ± 0.06c	7.26 ± 0.05c
Ala	BDL	BDL	BDL	BDL	BDL	47.640 ± 0.21b	48.507 ± 0.19a
Cys	4.22 ± 0.09a	4.04 ± 0.07b	3.99 ± 0.04b	4.12 ± 0.08a	4.24 ± 0.05a	2.05 ± 0.03c	1.90 ± 0.02c
Val	47.23 ± 0.19a	46.28 ± 0.12a	46.84 ± 0.17a	45.70 ± 0.11b	46.08 ± 0.20a	24.83 ± 0.09c	26.08 ± 0.14c
Met	6.18 ± 0.05a	5.82 ± 0.08a	5.68 ± 0.03a	5.62 ± 0.06a	5.87 ± 0.09a	2.94 ± 0.02b	2.96 ± 0.02b
Ile	24.65 ± 0.21a	24.02 ± 0.23a	24.97 ± 0.25a	22.36 ± 0.21b	22.79 ± 0.14b	11.77 ± 0.06c	12.04 ± 0.04c
Leu	BDL	BDL	BDL	24.82 ± 0.24a	BDL	13.40 ± 0.03b	13.47 ± 0.07b
Tyr	20.73 ± 0.15a	20.23 ± 0.13a	20.38 ± 0.16a	20.38 ± 0.09a	20.73 ± 0.08a	9.54 ± 0.09b	9.82 ± 0.07b
Phe	30.39 ± 0.22a	28.95 ± 0.19b	29.76 ± 0.21a	30.21 ± 0.20a	30.89 ± 0.17a	13.94 ± 0.06c	14.57 ± 0.03c
Lys	49.46 ± 0.27a	48.55 ± 0.24b	50.21 ± 0.26a	50.93 ± 0.25a	49.80 ± 0.21a	27.87 ± 0.04d	30.07 ± 0.07c
NH3	34.52 ± 0.25d	48.38 ± 0.29a	29.24 ± 0.14e	37.96 ± 0.11c	41.65 ± 0.25b	17.46 ± 0.08f	13.70 ± 0.11f
His	34.21 ± 0.19a	32.37 ± 0.17b	33.96 ± 0.21a	35.07 ± 0.15a	34.85 ± 0.20a	16.65 ± 0.09c	17.85 ± 0.06c
Arg	27.82 ± 0.17b	27.15 ± 0.15b	31.22 ± 0.19a	32.38 ± 0.16a	30.96 ± 0.22a	13.45 ± 0.10c	14.71 ± 0.12c
Total	312.19 ± 0.34e	331.97 ± 0.39d	314.36 ± 0.31e	350.60 ± 0.26c	335.59 ± 0.13d	382.98 ± 0.20b	398.68 ± 0.23a

Note

Outcomes with distinct letters in the same row (a–g) are significantly (*p *< .05) different from each other. PEF 2‐25, treated at 2 kV/cm with 25 pulses; PEF 2‐50, treated at 2 kV/cm with 50 pulses; PEF 4‐25, treated at 4 kV/cm with 25 pulses; PEF 4‐50, treated at 4 kV/cm with 50 pulses; PEF 6‐25; treated at 6 kV/cm with 25 pulses; PEF 6‐50, treated at 6 kV/cm with 50 pulses.

### 3.9 Effect on minerals composition

The results of elemental analysis of the untreated and PEF‐treated seeds plantlets are shown in Table 4. The elemental analysis indicated variations in mineral concentration in plantlets, according to treatment conditions. According to the obtained results, Ca, Zn, and Mn were significantly higher in PEF6‐50 than the untreated and other PEF‐treated seed plantlets, while Fe and K were lower as compared to the other PEF‐treated seed plantlets. Zn and Ca increased by 20.45% and 15.55%, with increasing PEF intensities PEF6‐50 as compared to untreated seed plantlets. Earlier, the Ca and Mg contents of wheat plantlets were more in those plantlets cultivated just on water (Kulkarni et al., 2006) and K, Mn, Zn, and Fe level increased in those plantlets cultivated in soil (Kulkarni, Acharya, Rajurkar, & Reddy, 2007). The fundamental stress differences of soil and hydroponic growth to release elements influenced by soil medium, as the exchange cation complex and the outward conditions of soil, could change the availability of the elements. The interaction of seed and water and PEF polarized the seeds, could influence to extract the elements from water and soil (Ade‐Omowaye, Angersbach, Eshtiaghi, & Knorr, 2000). Mn is a micronutrient that serves as the enzyme activator; it is the constitution of some enzymes. Mn is related to photosynthesis, oxygen‐producing reactions, water splitting, and structural improvement of the chloroplast lamellae (Shenker, Plessner, & Tel‐Or, 2004). The PEF polarized seeds have rapid development, so the initial manganese uptake was quick, via xylem pathways; that is why the high PEF‐treated seeds plantlets had higher manganese as compared to untreated seeds plantlets. The Mn had a positive interaction with Ca; in the current study, PEF‐treated seeds increased the Ca and Mn in plantlets (Millaleo, Reyes‐Díaz, Ivanov, Mora, & Alberdi, 2010). The results of K show that the increase of Mn reduces the level of K, and the PEF treatment enhances Mn, and ultimately photosynthesis due to large leaf area. High PEF‐treated seeds showed similarity for the K level with untreated seed plantlets. The Ca, Mn, Zn, and Fe are divalent, and the uptake of these minerals is less rapid than others, such as K is more readily available. The PEF creates stress that improves permeabilization water uptake, germination, vigor index, and overall plant growth due to facilitated the seeds and enhanced the nutrients uptake in quite systematic way (Vorobiev & Lebovka, 2009). The world population has a diet deficient in Fe (60%), Zn (30%), Ca, Mn, and K, so treatment of raw material such as plantlets is a very effective way to fulfill these deficiencies (Martínez‐Ballesta et al., 2010). Wheat plantlets juice consumption may contribute toward meeting daily mineral requirements.

**TABLE 4:** Minerals (mg/L) contents of PEF‐treated and untreated wheat seed plantlets

Minerals	Untreated	PEF 2‐25	PEF 2‐50	PEF 4‐25	PEF 4‐50	PEF 6‐25	PEF 6‐50
Zinc (Zn)	4.94 ± 1.3^cd^	5.11 ± 1.2^c^	5.53 ± 1.4^b^	5.60 ± 1.2^b^	5.96 ± 1.4^ab^	6.10 ± 1.3^a^	6.21 ± 1.4^a^
Iron (Fe)	12.4 ± 1.6^d^	15.5 ± 1.7^c^	23 ± 1.9^b^	27.6 ± 2.1^a^	16.7 ± 1.7^c^	9.75 ± 1.6^e^	11.4 ± 1.5^d^
Manganese (Mn)	2.83 ± 1.1^d^	3.06 ± 1.3^bc^	3.04 ± 1.2^bc^	2.92 ± 1.1^d^	2.92 ± 1.5^d^	3.26 ± 1.4^b^	3.65 ± 1.5^a^
Calcium (Ca)	190 ± 4.1^cd^	196 ± 3.6^c^	206 ± 3.5^b^	206 ± 3.1^b^	209 ± 3.9^b^	208 ± 3.3^b^	225 ± 3.4^a^
Potassium (K)	1584 ± 4.3^e^	1791 ± 5.2^a^	1711 ± 5.4^c^	1706 ± 4.1^c^	1,730 ± 5.5^b^	1703 ± 5.6^c^	1,610 ± 4.9^d^

Note

Outcomes with distinct letters in the same row (a–g) are significantly (*p *< .05) different from each other. PEF 2‐25, treated at 2 kV/cm with 25 pulses; PEF 2‐50, treated at 2 kV/cm with 50 pulses; PEF 4‐25, treated at 4 kV/cm with 25 pulses; PEF 4‐50, treated at 4 kV/cm with 50 pulses; PEF 6‐25, treated at 6 kV/cm with 25 pulses; PEF 6‐50, treated at 6 kV/cm with 50 pulses.

## 4 CONCLUSION

The present study demonstrates the use of PEF technology to manipulate a living plant system. In this study, the aim of the PEF application was to enhance the functional characteristics of wheat plantlets. The variations in seed water content influence the ability of a PEF treatment to induce significant and sustained changes in the metabolism of the resultant wheatgrass seedlings. PEF increased the activities of antioxidant enzymes in the resultant seedlings by stimulation and increasing the bioprotective capacity of harvested shoots. “Electro priming” of seeds using PEF, if appropriately optimized, could be used as a relatively simple method to produce high‐quality and nutritious sprouts without using chemical treatments. While the present study concentrated on the potential use of PEF treatment on imbibed seeds for the production of wheatgrass shoots with greater bioprotective capacity for human consumption, the resultant electro‐stimulated shoots could also be valuable food for human as well as a supplement for animal feed.

## CONFLICT OF INTEREST

The authors do not have any conflicts of interest.

## AUTHOR CONTRIBUTION

Zahoor Ahmed carried out the experimental work and Muhammad Faisal Manzoor did the preparation of the manuscript. Abdul Rahaman, Ume Roobab, Rabia Siddique, Abdul Qayyum, Zia ud Din, and Azhari Siddeeg helped in the results discussion and statistical analysis. Xin‐An Zeng and Nazir Ahmad revised the final paper.

## ETHICAL APPROVAL

This article does not contain any studies with human participants or animals performed by any of the authors.

## INFORMED CONSENT

For this type of study, formal consent is not required.
