# Corrigendum

**DOI:** 10.1002/fsn3.2627

**Published:** 2021-11-01

There were errors in the conclusion of Ahmed et al. (2020). The conclusion should have read:

The present study investigated the impact of PEF treatments on wheat seed development without imbibition (no water absorbed by seeds before treatment). The results revealed that the PEF strengths improve growth parameters including water uptake, germination rate, fresh and dry weight, vigor index A and B, total length, green and senescent leaves, and leaf area. Moreover, after juice extraction, the juice yield, soluble proteins, total phenolic, DPPH, amino acids, minerals, and pigments were significantly increased as compared to untreated samples. The outcome of the study verified that the PEF is a useful tool to improve the growth parameters of wheat seeds and also enhance the nutritional profile of wheat plantlets juice.
